# Association between preoperative anxiety states and postoperative complications in patients with esophageal cancer and COPD: a retrospective cohort study

**DOI:** 10.1186/s12885-024-11884-9

**Published:** 2024-05-17

**Authors:** Yu Rong, Yanbing Hao, Dong Wei, Yanming Li, Wansheng Chen, Li Wang, Tian Li

**Affiliations:** 1 Department of Thoracic Surgery, The First Affiliated Hospital of Hebei North University, 12 Changqing Road, 075000 Zhangjiakou, China; 2 Department of Anesthesiology, The First Affiliated Hospital of Hebei North University, 075000 Zhangjiakou, China; 3 School of Basic Medicine, Fourth Military Medical University, 710032 Xi’an, China

**Keywords:** Chronic obstructive pulmonary disease, Anxiety, Esophageal neoplasms, Postoperative complications, Retrospective study

## Abstract

**Background:**

Esophageal cancer brings emotional changes, especially anxiety to patients. Co-existing anxiety makes the surgery difficult and may cause complications. This study aims to evaluate effects of anxiety in postoperative complications of esophageal cancer patients with chronic obstructive pulmonary disease **(**COPD).

**Methods:**

Patients with esophageal cancer and co-existing COPD underwent tumor excision. Anxiety was measured using Hospital Anxiety and Depression Scale (HAD) before surgery. Clavien-Dindo criteria were used to grade surgical complications. A multiple regression model was used to analyze the relationship between anxiety and postoperative complications. The chi-square test was used to compare the differences in various types of complications between the anxiety group and the non-anxiety group. A multinomial logistic regression model was used to analyze the influencing factors of mild and severe complications.

**Results:**

This study included a total of 270 eligible patients, of which 20.7% had anxiety symptoms and 56.6% experienced postoperative complications. After evaluation by univariate analysis and multivariate logistic regression models, the risk of developing complications in anxious patients was 4.1 times than non-anxious patients. Anxious patients were more likely to develop pneumonia, pyloric obstruction, and arrhythmia. The presence of anxiety, surgical method, higher body mass index (BMI), and lower preoperative oxygen pressure may increase the incidence of minor complications. The use of surgical methods, higher COPD assessment test (CAT) scores, and higher BMI may increase the incidence of major complications, while anxiety does not affect the occurrence of major complications (*P* = 0.054).

**Conclusion:**

Preoperative anxiety is associated with postoperative complications in esophageal cancer patients with co-existing COPD. Anxiety may increase the incidence of postoperative complications, especially minor complications in patient with COPD and esophageal cancer.

## Introduction

With the rapid advance of medical technology, the theranostics of cancer patients has significantly improved [1]. However, a neoplastic pathological report usually means “death penalty” and trigger strong emotional changes [2]. Research on the psychological distress of cancer patients had already begun in the 1980s [3]. Anxiety is a type of mental disorder, and a nationwide epidemiological study in China reported that anxiety is the most common type of mental disorder [4]. Among cancer patients, anxiety is an emotional response to uncertainty, distress, and the threat of death., which are due to the uncertainty of therapeutic outcomes, fear of pain, and the possibility of death [5]. Indeed, anxiety has a motivating effect on patients to endure cancer treatment despite potential pain. However, it can also lead to a decrease in quality of life, compliance with treatment, and increased hospital stays and disability rates [6].

Globally, in 2020, the age-standardized incidence and mortality rate were 6.3 cases and 5.6 cases per 100,000 people, respectively [7]. Research has found that anxiety may be an pivotal factor contributing to the incidence of esophageal cancer [8]. Among patients who have already developed esophageal cancer, most of them are already in advanced stages when seeking medical attention. Surgical treatment is still the main treatment method for esophageal cancer patients at present [9]. However, only one-third of patients have the opportunity to receive surgical treatment. Anxiety may be due to the heavy burden of medical expenses, obvious difficulties in eating, fear of surgical risks, as well as restrictive and absorptive changes in gastrointestinal physiology and various postoperative complications [10–12].

Chronic obstructive pulmonary disease (COPD), a disease characterized by irreversible expiratory airflow limitation [13], is an independent risk factor for postoperative pulmonary complications in esophageal cancer [14]. As the global aging population accelerates, the proportion of esophageal cancer patients with COPD will further increase [15]. The prevalence of anxiety is high among COPD patients, with a review indicating that 10–90% of COPD patients experience anxiety [16]. However, previous studies rarely investigated the impact of anxiety on postoperative complications in patients with esophageal cancer and COPD. Therefore, we conducted this study to evaluate whether anxiety would have an impact on the occurrence and severity of postoperative complications in patients with esophageal cancer complicated by COPD, aiming to provide better guidance for the perioperative management.

## Method

### Study design

This study retrospectively reviewed patients with esophageal cancer who underwent surgical treatment in Department of Thoracic, First Affiliated Hospital of Hebei North University between Jan 2010 to Dec 2018. The study was approved by the ethics committee of the First Affiliated Hospital of Hebei North University (K2018075). The inclusion criteria for patients were: (1) postoperative pathology suggests squamous cell carcinoma; (2) exclusion of other organ metastasis by imaging examination; (3) pathological staging ranging from stage IA to IVA. The exclusion criteria were: (1) lack of pulmonary function test results; (2) history of other malignant tumors within five years; (3) FEV1/FVC > 70% after bronchodilator use; (4) non-curative surgery for esophageal cancer. None of the patients had received preoperative neoadjuvant therapy. The postoperative pathological staging of esophageal cancer was performed according to the eighth edition of the esophageal cancer staging system [17].

### Definition and measurement methods of variables

Anxiety was assessed using the Hospital Anxiety and Depression Scale (HAD) during routine evaluation upon admission. The evaluation period covered the patient’s emotional state in the past month. A score of 0–7 was considered as no anxiety, while a score greater than 7 indicated the presence of anxiety. If the patient was unable to read, the attending physician would read the content of the scale and ask the patient to make an assessment. The pulmonary function test was performed after the bronchodilator was inhaled. FEV1/FVC < 70% was used to classify the severity of chronic obstructive pulmonary disease according to the GOLD guidelines. Mild: FEV1 ≥ 80% predicted value, moderate: 50% predicted value ≤ FEV1 < 80% predicted value, severe: 30% predicted value ≤ FEV1 < 50% predicted value, very severe: FEV1 < 30% predicted value. The Clavien-Dindo classification system (CDC) was used to grade postoperative complications [18]. According to the level of treatment required for postoperative complications, they are divided into the following five grades: Grade I: no medication, surgery, endoscopy, radiation intervention or other treatments are required (use of antiemetics, analgesics, diuretics, electrolytes, and physical therapy is allowed). Grade II: other medications are required to treat Grade I complications. Grade IIIa: surgical, endoscopic or radiation treatment under local anesthesia. Grade IIIb: surgical, endoscopic or radiation treatment under general anesthesia. Grade IVa: single organ dysfunction, IVb: multiple organ dysfunction. Grade V: death. This includes both pulmonary and other postoperative complications, with Grade II and below being classified as mild complications, and Grade III and above being classified as severe complications [19]. The highest grade of complications experienced by a patient is recorded as the overall grade of that patient. Pneumonia is defined as a respiratory tract infection that requires antibiotic treatment, and is diagnosed if one or more of the following criteria are met: new onset of cough or change in the character of sputum, chest X-ray or computed tomography scan showing new infiltrates or worsening of existing infiltrates compared to previous images, fever (temperature > 38.0℃), and/or white blood cell count > 12 × 10^9^/L [20]. Bronchial asthma is defined as expiratory wheezing newly discovered after treatment with bronchodilators. Acute exacerbation of COPD is defined as worsening respiratory symptoms, increased sputum production, difficulty breathing, and asthma attacks compared to before [21]. Sepsis is defined as a clear infectious focus and meeting two or more of the following conditions: body temperature < 36 °C or > 38 °C; or heart rate > 90 beats/minute; or respiratory rate > 20 breaths/minute; or PaCO2 < 32mmHg; or white blood cell count < 4000/mm³ or > 12,000/mm³; or more than 10% immature neutrophils [22]. Acute respiratory distress syndrome (ARDS) is defined as arterial oxygen partial pressure (PaO2)/fraction of inspired oxygen (FiO2) < 200, positive end-expiratory pressure > 5 cm H_2_O, and duration > 24 h [23].

### Treatment methods and procedures

All patients underwent esophageal cancer resection and thoracic or cervical anastomosis. The choice of minimally invasive esophagectomy (MIE) or open esophagectomy (OE) was based on the preference of the patient or surgeon. In particular, MIE procedure typically employs McKeown procedure and Ivor-Lewis procedure, with the selection of procedure primarily contingent upon the tumor’s location in the patient. In the case of patients presenting with a tumor positioned at upper thoracic esophagus, McKeown procedure is typically employed to perform an anastomosis at the cervical region. This approach is undertaken to enhance the likelihood of achieving a greater negative rate at the esophageal margin. Conversely, for patients with a tumor located at a lower level, Ivor-Lewis procedure is more frequently chosen as it allows for the preservation of a longer esophagus and a reduction in the occurrence of postoperative reflux. A two-field lymphadenectomy were performed for all patients. The lymph node dissection performed during minimally invasive esophagectomy (MIE) encompasses a comprehensive range of lymph nodes, including those located in the thoracic region (such as the left and right recurrent laryngeal nerve, paraesophageal, paratracheal, subcarinal, supradiaphragmatic, and posterior mediastinal lymph nodes) as well as those in the abdominal region. All patients were routinely admitted to the intensive care unit to stabilize their condition and remove the tracheal tube after surgery. Patients whose symptoms were stable were transferred back to the general ward on 1st day. Patient-controlled analgesia with a pain pump was used for postoperative pain management. If the patient’s condition is stable, electrolyte-containing fluids can be administered through a gastric or jejunal nutrition tube 48 h after surgery. If there is no abdominal pain or abnormal drainage from the closed chest drainage tube, enteral nutrition solution can be given 96 h after surgery. When the patient is able to consume liquid diet and there is no obvious food residue in the drainage from the chest drainage tube, and the daily amount of drainage is less than 200 ml, removal of the chest drainage tube can be considered for discharge preparation. After discharge, supplementary feeding was continued through a gastric or jejunal nutrition tube.

### Statistical analysis

Continuous variables are expressed as mean ± standard deviation or median (interquartile range). Categorical variables are expressed as frequency (%). T-test is used to compare the differences between continuous variables with normal distribution and equal variances. Chi-square test is used to compare the differences between categorical variables. Whether to adjust for covariates is based on the following two criteria: the regression coefficient *p*-value of the covariate on the outcome variable is < 0.10, or introducing the covariate into the basic model leads to a change in the regression coefficient of the risk factor of more than 10% [24]. We used a binary logistic regression model to assess the relationship between anxiety and postoperative complications. Three models were used, adjusting for confounding variables that may affect the association between anxiety and postoperative complications in a stepwise manner. Model 1 was unadjusted, model was 2 adjusted for demographic parameters in model 1: gender (male, female), age (continuous), and model 3 was adjusted for Body mass index (BMI), COPD Assessment Test (CAT) score, preoperative arterial oxygen pressure (PaO_2_), Surgical procedure (MIE and OE), FEV1 as a percentage of predicted value (FEV1% Predicted), smoking index, and tumor staging based on model 2. Multinomial logistic regression was used to analyze the factors affecting minor and major postoperative complications. Data were analyzed using the statistical packages R (The R Foundation; http://www.r-project.org; version 4.2.0 2022-04-22), EmpowerStats (R) (www.empowerstats.com, X&Y Solutions, inc. Boston MA), and SPSS 26.0 (IBM Corp). All tests were conducted at a two-sided significance level of *P* < 0.05.

## Result

### Baseline data of patients

**Fig. 1:**
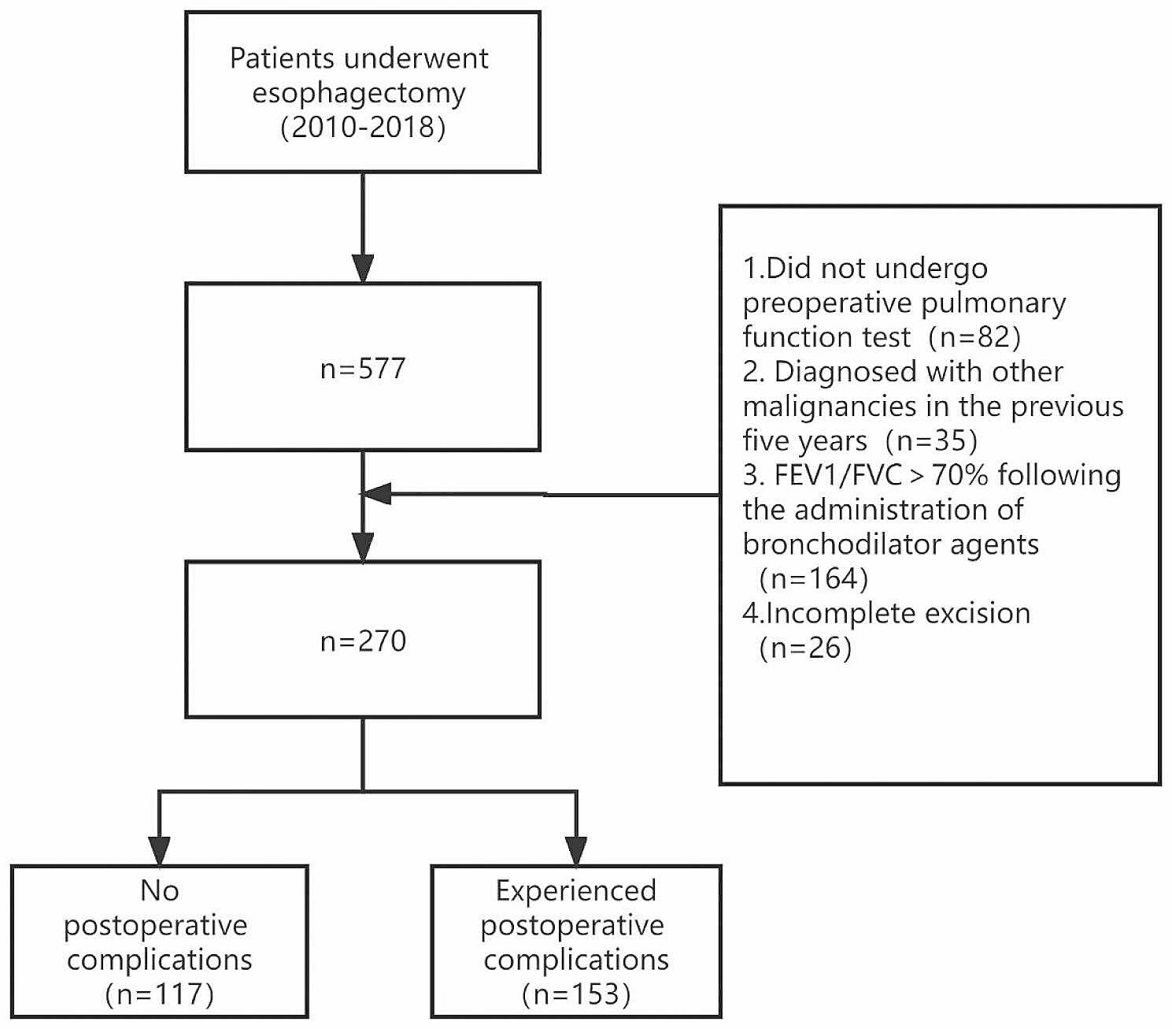
Study Flow Diagram

A total of 577 patients underwent radical esophagectomy during this period. Excluding 82 patients who did not undergo preoperative pulmonary function tests, 35 patients who developed other malignant tumors within five years, 164 patients without COPD, and 26 patients who underwent palliative resection, a total of 270 (242 males) eligible patients were finally included in the study. The mean age was 62.8 ± 8.6 years. There were 56 patients with anxiety (20.7%), and a total of 153 patients (56.6%) experienced postoperative complications. The patients’ mean BMI was 20.9 ± 2.2, mean left ventricular ejection fraction was 61.7 ± 4.2, and 132 patients underwent minimally invasive surgery (48.9%). The age-corrected comorbidity index was 3.3 ± 1.1, and tumor staging was as follows: stage I: 83 (30.7%), stage II: 73 (27.0%), stage III: 94 (34.8%), stage IV: 20 (7.4%). The baseline data of the patients are summarized in Table 1. The study flow chart is presented in Fig. 1.

**Table 1:** Baseline clinical data and patient characteristics

Characteristic	Non-anxiety	Anxiety	*P* value
Male (%)	192 (89.7%)	50 (89.3%)	0.924
Age, yr	62.8 ± 8.7	62.8 ± 8.3	0.984
Body mass index, kg/m^2^	20.9 ± 2.1	21.0 ± 2.4	0.882
Hypertension	35 (16.4%)	9 (16.1%)	0.959
Diabetes mellitus	28 (13.1%)	8 (14.3%)	0.814
CAT score	11.8 ± 3.1	13.3 ± 3.8	0.003
LVEF, %	61.7 ± 4.3	61.9 ± 3.6	0.821
PaO2, mmHg	80.4 ± 10.1	78.7 ± 10.5	0.276
PaCO2, mmHg	37.8 ± 2.9	38.4 ± 4.1	0.216
preoperative albumin concentration, g/L	41.2 ± 3.4	41.7 ± 3.4	0.320
Surgical Procedure (%)			0.626
OE group	111 (51.9%)	27 (48.2%)	
MIE group	103 (48.1%)	29 (51.8%)	
Reconstruction route			0.122
Cervical	105 (49.1%)	21 (37.5%)	
Intrathoracic	109 (50.9%)	35 (62.5%)	
Preoperative pulmonary function			
FVC, L	2.8 ± 0.5	2.8 ± 0.5	0.561
FEV1, L	1.8 ± 0.3	1.7 ± 0.4	0.251
FEV1/FVC, %	63.2 ± 5.8	61.9 ± 6.5	0.161
FEV1% predicted, %	66.8 ± 7.4	62.7 ± 7.9	< 0.001
alcohol consumption (%)			0.008
Never	44 (20.6%)	14 (25.0%)	
Previously	34 (15.9%)	18 (32.1%)	
Still drinking alcohol	136 (63.6%)	24 (42.9%)	
Smoking (%)			0.538
Never	10 (4.7%)	1 (1.8%)	
Previously	31 (14.5%)	10 (17.9%)	
Still smoking	173 (80.8%)	45 (80.4%)	
Smoking Index	747.8 ± 445.3	916.6 ± 605.2	0.020
Tumor location			0.223
Upper	48 (22.4%)	10 (17.9%)	
Middle	114 (53.3%)	26 (46.4%)	
Lower	52 (24.3%)	20 (35.7%)	
TNM Stage (%)			0.971
I	66 (30.8%)	17 (30.4%)	
II	58 (27.1%)	15 (26.8%)	
III	75 (35.0%)	19 (33.9%)	
IV	15 (7.0%)	5 (8.9%)	

The value is presented as n (%) or mean ± standard deviation

Abbreviations: CAT score, COPD assessment test score; LVEF, left ventricular ejection fraction; PaO2, partial pressure of oxygen; PaCO2, partial pressure of carbon dioxide; OE group, open esophagectomy group; FVC, Forced Vital Capacity; FEV1, Forced Expiratory Volume

### Relationship between anxiety and postoperative complications

Table 2 shows the results of the univariate analysis. These results suggest that BMI, CAT score, surgical procedures, FEV1 as a percentage of predicted, anxiety, and smoking index may be associated with the occurrence of postoperative complications. In contrast, gender, age, left ventricular ejection fraction (LVEF), PaO_2_, preoperative CO2 pressure (PaCO2), preoperative albumin concentration, FVC, FEV1, FEV1/FVC, alcohol consumption, smoking, and tumor staging are not significantly associated with the occurrence of complications. The results of the multivariate logistic regression are shown in Table 3, including the unadjusted model and the adjusted models. In the unadjusted model, the risk of developing complications in patients with anxiety was four times higher than that in non-anxious patients (OR: 4.0, 95% CI: 2.0 to 8.2, *P* < 0.001). In adjusted model 1 (adjusting for demographic characteristics: age, gender), the OR was 4.1 (95% CI: 2.0 to 8.3, *P* < 0.001). In adjusted model 2 (fully adjusted model), the risk of developing complications in anxious patients was 4.1 times higher than that in non-anxious patients (OR: 4.1, 95% CI: 1.9 to 8.9, *P* < 0.001).

**Table 2:** Univariate analysis of factors affecting postoperative complications

	OR (95%CI)	*P* value
Gender		
Female	1.0	
Male	1.6 (0.7, 3.5)	0.251
Age	1.0 (1.0, 1.0)	0.817
BMI	1.2 (1.0, 1.3)	0.013
Anxiety		
None	1.0	
Yes	4.0(2.0, 8.2)	< 0.001
CAT score	1.1 (1.1, 1.2)	0.001
LVEF	1.0 (1.0, 1.1)	0.678
PaO2	1.0 (1.0, 1.0)	0.096
PaCO2	1.0 (0.9, 1.0)	0.209
Preoperative albumin concentration	1.0 (0.9, 1.0)	0.470
Surgical Procedure		
OE group	1.0	
MIE group	0.4 (0.3, 0.7)	< 0.001
Preoperative pulmonary function		
FVC	0.9 (0.5, 1.5)	0.695
FEV1	0.8 (0.4, 1.6)	0.506
FEV1/FVC	1.0 (0.9, 1.0)	0.343
FEV1% predicted	1.0 (0.9, 1.0)	0.004
Alcohol consumption		
Never	1.0	
Previously	1.2 (0.6, 2.6)	0.621
Still drinking alcohol	0.9 (0.5, 1.7)	0.803
Smoking		
Never	1.0	
Previously	0.7 (0.2, 2.5)	0.531
Still smoking	1.2 (0.4, 4.1)	0.761
Smoking Index	1.0 (1.0, 1.0)	0.004
TNM stage		
I	1.0	
II	1.5 (0.8, 2.8)	0.226
III	1.4 (0.8, 2.5)	0.292
IV	1.5 (0.5, 4.0)	0.451

**Table 3:** Multiple regression analysis of anxiety on postoperative complications

Exposure	Non-adjusted	Adjust I	Adjust II
anxiety			
None	1.0	1.0	1.0
Yes	4.0 (2.0, 8.2) < 0.001	4.1 (2.0, 8.3) < 0.001	4.1 (1.9, 8.9) < 0.001

Non-adjusted model adjust for: None

Adjust I model adjust for: gender; age

Adjust II model adjust for: gender; age; BMI; CAT score; PaO2; Surgical Procedure; FEV1% predicted; Smoking Index; TNM stage;

### Types of complications and anxiety

Among the complications that occurred, the incidence rates in the group with combined anxiety were as follows: pneumonia (37.5%), arrhythmia (21.4%), atelectasis requiring bronchoscopy (19.6%), acute exacerbation of COPD (16.1%), pleural effusion requiring additional drainage (14.3%), anastomotic fistula (14.3%), wound infection (14.3%), recurrent laryngeal nerve injury (12.5%), pyloric obstruction (7.1%), asthma (7.1%), ARDS (7.1%), gastroparesis (5.4%), pneumothorax requiring re-intubation (3.6%), systemic sepsis (3.6%), heart failure (1.8%), and death (1.8%). The incidence rates in the group without anxiety were as follows: pneumonia (24.3%), atelectasis requiring bronchoscopy (13.6%), pleural effusion requiring additional drainage (12.6%), acute exacerbation of COPD (10.7%), recurrent laryngeal nerve injury (9.3%), anastomotic fistula (7.9%), wound infection (7.5%), arrhythmia (6.1%), asthma (5.6%), ARDS (5.6%), pneumothorax requiring re-intubation (3.7%), heart failure (3.7%), gastroparesis (2.8%), chylothorax (1.9%), pyloric obstruction (0.9%), systemic sepsis (0.9%), and death (0.9%). Table 4 shows the differences in the types of complications between the group with anxiety and without anxiety. Patients with anxiety were more likely to develop pneumonia (OR: 1.9, 95% CI: 1.0 to 3.5, *P* = 0.048), pyloric obstruction (OR: 8.2, 95% CI: 1.5 to 45.7, *P* = 0.022), and arrhythmia (OR: 4.2, 95% CI: 1.8 to 10.0, *P* < 0.001).

**Table 4:** The impact of anxiety on the types of postoperative complications

	Non-anxiety	Anxiety	OR (95%CI)	*P* value
Recurrent laryngeal nerve injury	20(9.3)	7(12.5)	1.4(0.6, 3.5)	0.484
Pneumonia	52(24.3)	21(37.5)	1.9(1.0, 3.5)	0.048
Anastomotic fistula	17(7.9)	8(14.3)	1.9 (0.8, 4.7)	0.145
Gastroparesis	6(2.8)	3(5.4)	2.0(0.5, 8.1)	0.596
Pyloric obstruction	2(0.9)	4(7.1)	8.2(1.5, 45.7)	0.022
Arrhythmia requiring intervention	13(6.1)	12(21.4)	4.2(1.8, 10.0)	< 0.001
Congestive heart failure requiring intervention	8(3.7)	1(1.8)	0.5(0.1, 3.8)	0.759
Bronchial asthma	12(5.6)	4(7.1)	1.3(0.4, 4.2)	0.908
Acute exacerbation of COPD	23(10.7)	9(16.1)	1.6(0.7, 3.7)	0.272
Thoracic incision dehiscence	16(7.5)	8(14.3)	2.1(0.8, 5.1)	0.183
Chylothorax	4(1.9)	0(0.0)	-	0.583
Pulmonary atelectasis requiring bronchoscopy	29(13.6)	11(19.6)	1.6(0.7, 3.4)	0.253
Pneumothorax requiring reintubation	8(3.7)	2(3.6)	1.0(0.2, 4.6)	1.000
Pleural effusion requiring additional drainage	27(12.6)	8(14.3)	1.2(0.5, 2.7)	0.741
ARDS	12(5.6)	4(7.1)	1.3(0.4, 4.2)	0.908
Systemic sepsis	2(0.9)	2(3.6)	3.9 (0.5, 28.5)	0.191
death	2(0.9)	1(1.8)	1.9(0.2, 21.6)	0.504

### Severity of complications and anxiety

**Fig. 2:**
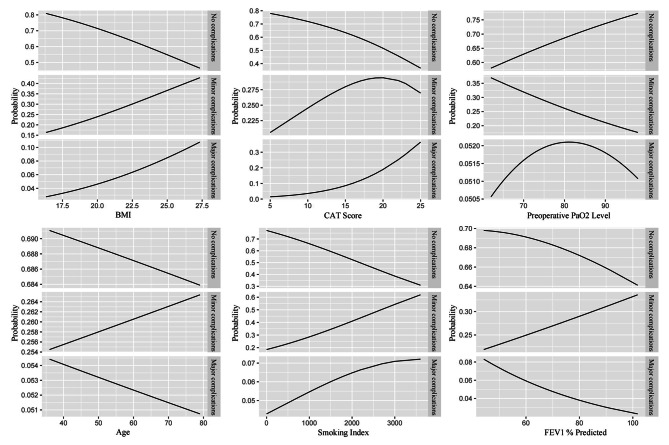
The predictive results of each continuous independent variable on the severity of complications

**Fig. 3:**
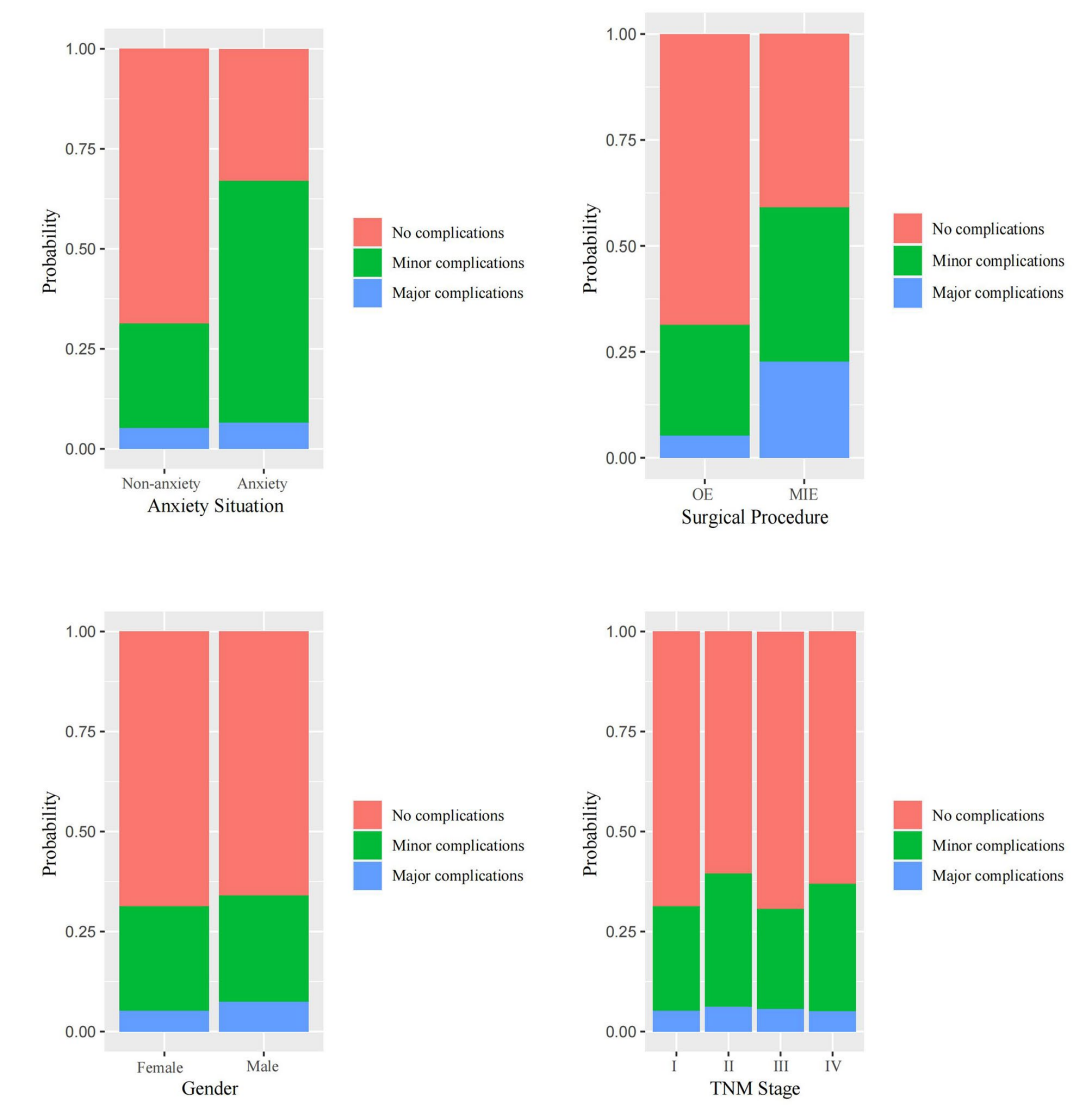
The predictive results of each categorical independent variable on the severity of complications

Among all 153 patients who experienced complications, there were 98 cases of minor complications, of which 32 cases (32.6%) had anxiety; 55 cases of major complications, of which 13 cases (23.6%) had anxiety. The results of the multinomial logistic regression (Table 5) showed that compared with patients without complications, the presence of anxiety (OR: 4.8, 95% CI: 2.2 to 10.6, *P* < 0.001), the use of OE procedure (OR: 2.3, 95% CI: 1.3 to 4.4, *P* = 0.007), higher BMI (OR: 1.1, 95% CI: 1.0 to 1.3, *P* = 0.041), and lower PaO_2_ (OR: 1.0, 95% CI: 0.9 to 1.0, *P* = 0.041) may increase the occurrence of minor complications. The use of OE procedure (OR: 7.3, 95% CI: 3.2 to 16.6, *P* < 0.001), higher CAT scores (OR: 1.2, 95% CI: 1.1 to 1.4, *P* = 0.007), and higher BMI (OR: 1.2, 95% CI: 1.0 to 1.4, *P* = 0.034) may increase the occurrence of major complications, while anxiety does not affect the occurrence of major complications (*P* = 0.054). The predictive results of each variable for the severity of complications are shown in Figs. 2 and 3.

**Table 5:** Multinomial logistics regression of factors affecting the severity of complications

	No complications	Minor complications		Major complications	
		OR (95% CI)	*P* value	OR (95% CI)	*P* value
(Intercept)	1.0 (ref.)	0.1(0.1, 0.1)	< 0.001	0.0 (0.0, 0.0)	< 0.001
Male	1.0 (ref.)	1.1 (0.4, 2.7)	0.913	1.5 (0.4, 5.9)	0.572
Age	1.0 (ref.)	1.0 (1.0, 1.0)	0.944	1.0 (1.0, 1.0)	0.947
BMI	1.0 (ref.)	1.1 (1.0, 1.3)	0.041	1.2(1.0, 1.4)	0.034
Anxiety	1.0 (ref.)	4.8 (2.2, 10.6)	< 0.001	2.6 (1.0, 7.0)	0.054
CAT Score	1.0 (ref.)	1.1 (0.9, 1.2)	0.413	1.2 (1.1, 1.4)	0.007
PaO2	1.0 (ref.)	1.0 (0.9, 1.0)	0.041	1.0 (1.0, 1.0)	0.664
OE group	1.0 (ref.)	2.3 (1.3, 4.4)	0.007	7.3 (3.2, 16.6)	< 0.001
FEV1% predicted	1.0 (ref.)	1.0 (1.0, 1.1)	0.685	1.0 (0.9, 1.0)	0.483
Smoking Index	1.0 (ref.)	1.0 (1.0, 1.0)	0.183	1.0 (1.0, 1.0)	0.426
TNM Stage					
II	1.0 (ref.)	1.4 (0.7, 3.1)	0.341	1.4 (0.5, 3.6)	0.548
III	1.0 (ref.)	0.9 (0. 5, 2.0)	0.883	1.1 (0.4, 2.6)	0.887
IV	1.0 (ref.)	1.3 (0.4, 4.6)	0.659	1.1 (0.2, 5.2)	0.930

## Discussion

### Main finding and interpretation

In this study, we found a significant correlation between anxiety and postoperative complications in patients with esophageal cancer combined with COPD. Neoplasms remain the main chronic diseases worldwide [25–30]. The results suggest that anxiety is a contributing factor to the occurrence of postoperative complications. After controlling for other variables, the likelihood of postoperative complications in patients with anxiety was approximately 4.1 times higher than in patients without anxiety. Among the types of complications, the incidence of postoperative pneumonia, arrhythmia, and pyloric obstruction was higher in anxious patients than non-anxious patients. Compared to patients without complications, anxiety increased the incidence of mild postoperative complications.

A study on the complications related to esophagectomy using an internationally standardized dataset showed that the overall incidence of postoperative complications after esophagectomy was approximately 59%, with pneumonia being the most common complication among all [31]. Our study results are similar to those of the standardized study. Approximately one-fifth of patients in previous studies were found to have anxiety, while the proportion of anxiety in esophageal cancer patients found in previous studies was even higher, accounting for as much as one-fourth or more [32, 33]. One possible reason for this difference is that our study did not include late-stage patients who were no longer eligible for surgery, while late-stage cancer patients often have a shorter survival time, more obvious symptoms, and are more likely to experience anxiety [34–36]. Another possible reason is that different cultural backgrounds may lead to differences in the perception of emotional states. For example, some studies have shown that anxiety levels in Asian populations tend to be lower than those in non-Asian populations [37, 38].

In the 1980s, researchers began to pay attention to the impact of preoperative anxiety on postoperative recovery [39, 40]. These studies have shown that preoperative anxiety may lead to delayed postoperative recovery and increased incidence of complications. Measures such as preoperative decompression and sedatives have been used to alleviate patients’ anxiety in order to better promote postoperative recovery. Research suggests that anxiety is a contributing factor to postoperative complications [41]. This is consistent with our research findings. One reason is that preoperative psychological factors can affect physiological functions. Anxiety can cause overactivation of the sympathetic nervous system, which in turn leads to changes in the secretion levels of hormones such as cortisol and catecholamines [42–44]. The consequences of these elevated hormones include suppression of the immune system, making patients more susceptible to postoperative complications such as wound infection, anastomotic fistula, and pneumonia [45]. In addition, patients with preoperative anxiety require higher doses of sedatives to achieve adequate levels of sedation [46], and higher doses of sedatives are closely related to postoperative nausea, vomiting, and cardiorespiratory complications [47].

Unlike other studies, all patients included in our study had COPD. The incidence of anxiety is higher in COPD patients, and the incidence of postoperative complications is significantly increased [48–52]. The results of these studies are consistent with the findings observed in our study. Therefore, anxiety may have an impact on the occurrence of postoperative complications in patients through the pathways mentioned above. As we found in our research, pneumonia is the most common complication with the highest incidence rate. Firstly, due to the longer duration of esophageal cancer surgery, a larger amount of fluid (including colloidal fluids and blood transfusions) is administered during the operation, which increases the load on the pulmonary circulation and makes it prone to postoperative pneumonia [53]. Secondly, airway is governed by the autonomic nervous system, which provides continuous control over the smooth muscle, secretory cells, and vascular system of the airway [54, 55]. The autonomic nervous system consists of the sympathetic and parasympathetic nervous systems. The parasympathetic nervous system is innervated by the left and right vagus nerves running in the posterior mediastinum between the trachea and esophagus. In clinical practice, some inhaled drugs that alter the activity of autonomic nervous system receptors, including anticholinergic agents and beta-adrenergic agonists, are the main medications for treating COPD. Under normal circumstances, the vagus nerve prevents lung overinflation by participating in the cough reflex and Hering-Breuer reflex [56]. At the same time, the pulmonary C-fibers (PCFs) in the vagus nerve play a crucial role in sensing and responding to lung infections and inflammatory cytokines [57]. The vagus nerve is a major component of the parasympathetic division of the autonomic nervous system. Anxiety can cause widespread activation of the sympathetic nervous system [58]. Therefore, under the comprehensive impact of the above mechanisms, it is possible to significantly increase the incidence of pneumonia Our research has found that cardiac arrhythmia is another postoperative complication that exists differently due to anxiety. This is consistent with previous studies that have found anxiety to be an independent risk factor for cardiac arrhythmia [59]. Pyloric obstruction is a common postoperative complication in the digestive tract, with one cause being surgical operation [60]. On the other hand, weakened vagal nerve activity in anxious patients may also be a possible cause of postoperative pyloric obstruction [61].

As far as we know, this is currently the largest study on the relationship between emotional status and postoperative complications in esophageal cancer patients with COPD. It is also the first to confirm that anxiety increases the incidence of postoperative complications in this patient population. This study highlights the need for clinical doctors to pay more attention to anxiety as a commonly overlooked preoperative emotional status that may require more intervention.

### Limitations

However, there are some limitations to this study. Firstly, it lacks sociodemographic data on patients, such as their education level, income, and place of residence, which may also be factors affecting patient anxiety. Future studies could include this type of information. Secondly, this study is a retrospective study. Although multivariate regression can adjust for measured covariates, it cannot account for potential residual confounding effects. Finally, the study population was limited to esophageal cancer patients with COPD, and the results may not necessarily apply to other populations.

## Conclusion

Although there are limitations as mentioned above, our research provides further support that preoperative anxiety could be associated with postoperative complications in esophageal cancer patients with co-existing COPD. Anxiety may lead to an increased incidence of postoperative complications, especially minor complications, in this population. These complications mainly include pneumonia, pyloric obstruction, and arrhythmia.

## Data Availability

Data are available from corresponding author upon reasonable requests.

## Abbreviations

COPD: Chronic Obstructive Pulmonary Disease
HAD: Hospital Anxiety and Depression Scale
CDC: Clavien-Dindo classification system
FVC: Forced Vital Capacity
FEV1: Forced Expiratory Volume in one second
CAT: COPD Assessment Test
BMI: Body Mass Index
OR: Odds ratios
CI: Confidence interval

## References

[1] Mullard A (2020). Addressing cancer’s grand challenges. Nat Rev Drug Discov.

[2] Tan SM, Beck KR, Li H, Lim EC, Krishna LK (2014). Depression and anxiety in cancer patients in a Tertiary General Hospital in Singapore. Asian J Psychiatr.

[3] Derogatis LR, Morrow GR, Fetting J (1983). The prevalence of psychiatric disorders among cancer patients. JAMA.

[4] Huang Y, Wang Y, Wang H (2019). Prevalence of mental disorders in China: a cross-sectional epidemiological study. Lancet Psychiatry.

[5] Traeger L, Greer JA, Fernandez-Robles C, Temel JS, Pirl WF (2012). Evidence-based treatment of anxiety in patients with cancer. J Clin Oncol.

[6] Berard RM. Depression and anxiety in oncology: the psychiatrist’s perspective. J Clin Psychiatry 2001 62 Suppl 8:58–61; discussion 2–3. 12108824

[7] Morgan E, Soerjomataram I, Rumgay H (2022). The Global Landscape of esophageal squamous cell carcinoma and esophageal adenocarcinoma incidence and mortality in 2020 and projections to 2040: new estimates from GLOBOCAN 2020. Gastroenterology.

[8] Zhu J, Zhou Y, Ma S (2021). The association between anxiety and esophageal cancer: a nationwide population-based study. Psychooncology.

[9] Obermannová R, Alsina M, Cervantes A (2022). Oesophageal cancer: ESMO Clinical Practice Guideline for diagnosis, treatment and follow-up. Ann Oncol.

[10] Chan RJ, Gordon LG, Tan CJ (2019). Relationships between Financial Toxicity and Symptom Burden in Cancer survivors: a systematic review. J Pain Symptom Manage.

[11] Verdonschot R, Baijens LWJ, Vanbelle S (2017). Affective symptoms in patients with oropharyngeal dysphagia: a systematic review. J Psychosom Res.

[12] Housman B, Flores R, Lee DS (2021). Narrative review of anxiety and depression in patients with esophageal cancer: underappreciated and undertreated. J Thorac Dis.

[13] Global strategy for the diagnosis, management, and prevention of chronic obstructive pulmonary disease (2021 report). In.; 2020: https://goldcopd.org/wp-content/uploads/2020/11/GOLD-REPORT-1-v1.1-25Nov20_WMV.pdf.

[14] Ohi M, Toiyama Y, Omura Y (2019). Risk factors and measures of pulmonary complications after thoracoscopic esophagectomy for esophageal cancer. Surg Today.

[15] Chiang CL, Hu YW, Wu CH (2016). Spectrum of cancer risk among Taiwanese with chronic obstructive pulmonary disease. Int J Clin Oncol.

[16] Hynninen KM, Breitve MH, Wiborg AB, Pallesen S, Nordhus IH (2005). Psychological characteristics of patients with chronic obstructive pulmonary disease: a review. J Psychosom Res.

[17] Rice TW, Ishwaran H, Ferguson MK, Blackstone EH, Goldstraw P (2017). Cancer of the Esophagus and Esophagogastric Junction: an Eighth Edition staging primer. J Thorac Oncol.

[18] Dindo D, Demartines N, Clavien PA (2004). Classification of surgical complications: a new proposal with evaluation in a cohort of 6336 patients and results of a survey. Ann Surg.

[19] Clavien PA, Barkun J, de Oliveira ML (2009). The Clavien-Dindo classification of surgical complications: five-year experience. Ann Surg.

[20] Ou X, Wang Q, Li C, Zhao H, Guo L. Magnetic Resonance Imaging Based on Wavelet Algorithm in the Diagnosis and Treatment of Tibial Osteomyelitis Wound Infection. Scientific Programming 2021, 2021:2130089.

[21] Rabe KF, Watz H (2017). Chronic obstructive pulmonary disease. The Lancet.

[22] Karalapillai D, Weinberg L, Peyton P (2020). Effect of intraoperative low tidal volume vs conventional tidal volume on postoperative pulmonary complications in patients undergoing major surgery: a Randomized Clinical Trial. JAMA.

[23] Matthay MA, Ware LB, Zimmerman GA (2012). The acute respiratory distress syndrome. J Clin Invest.

[24] Kernan WN, Viscoli CM, Brass LM (2000). Phenylpropanolamine and the risk of hemorrhagic stroke. N Engl J Med.

[25] Feng Y, Yang Y, Fan C (2016). Pterostilbene inhibits the growth of human esophageal Cancer cells by regulating endoplasmic reticulum stress. Cell Physiol Biochem.

[26] Hu W, Yang Y, Fan C (2016). Clinical and pathological significance of N-Myc downstream-regulated gene 2 (NDRG2) in diverse human cancers. Apoptosis.

[27] Li T, Yang Z, Jiang S (2018). Melatonin: does it have utility in the treatment of haematological neoplasms?. Br J Pharmacol.

[28] Ma Z, Fan C, Yang Y (2016). Thapsigargin sensitizes human esophageal cancer to TRAIL-induced apoptosis via AMPK activation. Sci Rep.

[29] Sun M, Liu X, Xia L (2021). A nine-lncRNA signature predicts distant relapse-free survival of HER2-negative breast cancer patients receiving taxane and anthracycline-based neoadjuvant chemotherapy. Biochem Pharmacol.

[30] Yang Z, Jiang S, Lu C (2019). SOX11: friend or foe in tumor prevention and carcinogenesis?. Ther Adv Med Oncol.

[31] Low DE, Kuppusamy MK, Alderson D (2019). Benchmarking complications Associated with Esophagectomy. Ann Surg.

[32] Bergquist H, Ruth M, Hammerlid E (2007). Psychiatric morbidity among patients with cancer of the esophagus or the gastro-esophageal junction: a prospective, longitudinal evaluation. Dis Esophagus.

[33] Dempster M, McCorry NK, Brennan E (2012). Psychological distress among survivors of esophageal cancer: the role of illness cognitions and coping. Dis Esophagus.

[34] Johanes C, Monoarfa RA, Ismail RI, Umbas R (2013). Anxiety level of early- and late-stage prostate cancer patients. Prostate Int.

[35] Meyer F, Fletcher K, Prigerson HG, Braun IM, Maciejewski PK (2015). Advanced cancer as a risk for major depressive episodes. Psychooncology.

[36] Xie Q, Sun C, Fei Z, Yang X (2022). Accepting Immunotherapy after Multiline Treatment failure: an exploration of the anxiety and depression in patients with Advanced Cancer Experience. Patient Prefer Adherence.

[37] Kalwar SK (2010). Comparison of human anxiety based on different cultural backgrounds. Cyberpsychol Behav Soc Netw.

[38] Guerrini CJ, Schneider SC, Guzick AG (2021). Psychological distress among the U.S. General Population during the COVID-19 pandemic. Front Psychiatry.

[39] Johnston M, Carpenter L (1980). Relationship between pre-operative anxiety and post-operative state. Psychol Med.

[40] Reading AE (1982). The effects of psychological preparation on pain and recovery after minor gynaecological surgery: a preliminary report. J Clin Psychol.

[41] Pan X, Wang J, Lin Z, Dai W, Shi Z (2019). Depression and anxiety are risk factors for Postoperative Pain-related symptoms and complications in patients undergoing primary total knee arthroplasty in the United States. J Arthroplasty.

[42] van Goozen SH, Matthys W, Cohen-Kettenis PT (1998). Salivary cortisol and cardiovascular activity during stress in oppositional-defiant disorder boys and normal controls. Biol Psychiatry.

[43] Poon JA, Turpyn CC, Hansen A, Jacangelo J, Chaplin TM (2016). Adolescent substance Use & psychopathology: interactive effects of Cortisol reactivity and emotion regulation. Cognit Ther Res.

[44] Ozbay F, Fitterling H, Charney D, Southwick S (2008). Social support and resilience to stress across the life span: a neurobiologic framework. Curr Psychiatry Rep.

[45] Pössel P, Ahrens S, Hautzinger M (2005). Influence of cosmetics on emotional, autonomous, endocrinological, and immune reactions. Int J Cosmet Sci.

[46] Maranets I, Kain ZN (1999). Preoperative anxiety and intraoperative anesthetic requirements. Anesth Analg.

[47] Liang Z, Gu Y, Duan X (2016). Design of multichannel functional near-infrared spectroscopy system with application to propofol and sevoflurane anesthesia monitoring. Neurophotonics.

[48] Wagena EJ, van Amelsvoort LG, Kant I, Wouters EF (2005). Chronic bronchitis, cigarette smoking, and the subsequent onset of depression and anxiety: results from a prospective population-based cohort study. Psychosom Med.

[49] Kim HJ, Lee J, Park YS (2016). Impact of GOLD groups of chronic pulmonary obstructive disease on surgical complications. Int J Chron Obstruct Pulmon Dis.

[50] Dai J, He Y, Maneenil K (2021). Timing of chronic obstructive pulmonary disease diagnosis in lung cancer prognosis: a clinical and genomic-based study. Transl Lung Cancer Res.

[51] Xu K, Cai W, Zeng Y (2021). Video-assisted thoracoscopic surgery for primary lung cancer resections in patients with moderate to severe chronic obstructive pulmonary diseases. Transl Lung Cancer Res.

[52] Xiao H, Zhou H, Liu K (2019). Development and validation of a prognostic nomogram for predicting post-operative pulmonary infection in gastric cancer patients following radical gastrectomy. Sci Rep.

[53] Russotto V, Sabaté S, Canet J (2019). Development of a prediction model for postoperative pneumonia: a multicentre prospective observational study. Eur J Anaesthesiol.

[54] Ikeda T, Anisuzzaman AS, Yoshiki H (2012). Regional quantification of muscarinic acetylcholine receptors and β-adrenoceptors in human airways. Br J Pharmacol.

[55] Undem BJ, Potenzieri C (2012). Autonomic neural control of intrathoracic airways. Compr Physiol.

[56] Belvisi MG (2002). Overview of the innervation of the lung. Curr Opin Pharmacol.

[57] Huang Y, Zhao C, Su X (2019). Neuroimmune regulation of lung infection and inflammation. QJM.

[58] Roth WT, Doberenz S, Dietel A (2008). Sympathetic activation in broadly defined generalized anxiety disorder. J Psychiatr Res.

[59] Carnevali L, Vacondio F, Rossi S (2015). Cardioprotective effects of fatty acid amide hydrolase inhibitor URB694, in a rodent model of trait anxiety. Sci Rep.

[60] Urschel JD, Blewett CJ, Young JE, Miller JD, Bennett WF (2002). Pyloric drainage (pyloroplasty) or no drainage in gastric reconstruction after esophagectomy: a meta-analysis of randomized controlled trials. Dig Surg.

[61] Greaves-Lord K, Ferdinand RF, Sondeijker FE (2007). Testing the tripartite model in young adolescents: is hyperarousal specific for anxiety and not depression?. J Affect Disord.

